# Prognostic Significance of Inflammatory Markers in Patients with Immune Thrombocytopenia

**DOI:** 10.3390/ijms27125528

**Published:** 2026-06-18

**Authors:** Nur Oğuz Davutoğlu, Ali İhsan Gemici, Merve Kocaköse, Selçuk Uylaş, Şeyma Tanır, Gökhan Pektaş, Mehmet Bilgehan Pektaş

**Affiliations:** 1 Division of Hematology, Eskişehir City Hospital, Eskişehir 26080, Türkiye; nuroguzz@hotmail.com; 2 Division of Hematology, Faculty of Medicine, Dokuz Eylül University, İzmir 35340, Türkiye; agemici21@yahoo.com; 3 Department of Internal Medicine, Faculty of Medicine, Muğla Sıtkı Koçman University, Muğla 48000, Türkiye; kocakosemerve@gmail.com (M.K.); selcukuylas@gmail.com (S.U.); seymatanir@mu.edu.tr (Ş.T.); 4 Division of Hematology, Faculty of Medicine, Muğla Sıtkı Koçman University, Muğla 48000, Türkiye; gokhanpektas@gmail.com; 5 Department of Medical Pharmacology, Faculty of Medicine, Afyonkarahisar Health Sciences University, Afyonkarahisar 03200, Türkiye

**Keywords:** immune thrombocytopenia, inflammation, platelet-to-lymphocyte ratio, D-dimer

## Abstract

Immune thrombocytopenia (ITP) is a heterogeneous autoimmune disorder characterized by immune-mediated platelet destruction and impaired platelet production. Increasing evidence suggests that systemic inflammation plays a significant role in disease pathogenesis and clinical outcomes. This study aimed to evaluate the prognostic significance of inflammatory indices and their association with complications, mortality, treatment response, and relapse in patients with ITP. In this single-center retrospective study, 166 adult patients diagnosed with primary ITP between January 2015 and December 2024 were analyzed. Demographic, clinical, and laboratory data at diagnosis were collected. Inflammatory indices derived from complete blood count parameters, including neutrophil-to-lymphocyte ratio (NLR) and platelet-to-lymphocyte ratio (PLR), were evaluated. Their associations with clinical outcomes were assessed using appropriate statistical methods. During the observation period based on retrospective medical records, complications occurred in 12% of patients, and mortality was observed in 6.6%. Patients with complications had significantly higher D-dimer levels and reduced bone marrow megakaryocyte production. In group comparisons, mortality was significantly associated with advanced age, male sex, and comorbidities. Laboratory findings revealed that lower hemoglobin, lymphocyte count, mean platelet volume, and albumin levels, along with higher PLR, erythrocyte sedimentation rate, bilirubin, and D-dimer levels, were significantly associated with mortality. Inflammatory indices such as NLR and PLR were not associated with complication development, but PLR was significantly associated with mortality. Response to intravenous immunoglobulin (IVIG) therapy was significantly associated with higher total protein, albumin, and fibrinogen levels, and lower erythrocyte sedimentation rate. Relapse was significantly associated in group comparisons with increased inflammatory activity, higher reticulocyte count, and positivity for antinuclear antibodies and Helicobacter pylori antigen. Systemic inflammation and impaired megakaryopoiesis play critical roles in the prognosis of ITP. While conventional inflammatory indices showed limited predictive value for complications, markers such as PLR, D-dimer, and albumin were associated with mortality and clinical outcomes. These findings suggest that readily available laboratory parameters may provide valuable insights for risk stratification and personalized management in patients with ITP.

## 1. Introduction

Immune thrombocytopenic purpura (ITP) is a heterogeneous autoimmune disorder characterized by a platelet count below 100 × 10^9^/L in the absence of identifiable secondary causes. In addition to impaired platelet production in the bone marrow, accelerated platelet destruction mediated by both humoral and cellular immune mechanisms is observed [1]. Although the precise pathophysiology of ITP has not yet been fully elucidated, immune dysregulation and genetic predisposition triggered by various factors—such as autoimmune conditions, infections, inflammatory states, and vaccinations—are known to contribute to its development [2,3]. The immune response directed against platelet autoantigens leads to enhanced splenic clearance of young platelets and reduced platelet production by bone marrow megakaryocytes. Autoantibodies specifically targeting platelet glycoproteins (GPs), including GPIIb/IIIa and GPIb/IX, are considered the primary drivers of both increased platelet destruction and insufficient platelet production [4]. Although ITP is widely recognized as an autoimmune disease, contemporary data regarding its association with systemic inflammation remain limited. Nevertheless, accumulating evidence in recent years suggests that inflammation may play a critical role in both the development and prognosis of ITP [5,6]. Inflammatory processes may exacerbate ITP by increasing immune cell populations such as neutrophils, lymphocytes, platelets, and monocytes [7]. Interactions among immune cells lead to spontaneous elevations in inflammatory and pro-inflammatory cytokines, including interleukin-10 (IL-10), IL-18, interferon-alpha (IFN-α), tumor necrosis factor-alpha (TNF-α), and IL-17, which are ultimately associated with enhanced immune responses [8,9,10]. The identification of inflammatory risk factors has therefore become increasingly important for the development of individualized treatment strategies in patients with ITP. In particular, the evaluation of cellular indices offers significant advantages over direct measurement of inflammatory cytokines and other intracellular markers, including lower cost and shorter turnaround time. Numerous studies have investigated the role of various cellular and non-cellular mediators, demonstrating that assessment of changes in their levels can aid in patient stratification and disease evaluation [11]. From this perspective, parameters and ratios derived from routine and readily accessible complete blood count analyses—such as the neutrophil-to-lymphocyte ratio (NLR), platelet-to-lymphocyte ratio (PLR), and platelet-to-neutrophil ratio (PNR)—have been proposed as markers of systemic inflammatory response and may be useful in determining the prognosis of certain diseases [12]. In the context of this study, systemic inflammation refers to a chronic, low-grade inflammatory state reflected by alterations in routine hematological and biochemical parameters rather than acute inflammatory responses.

## 2. Results

A total of 166 patients with immune thrombocytopenic purpura (ITP) were included in the study. The mean age at diagnosis was 53.5 years (range: 19–97 years), and 107 patients (64.4%) were female. A proportion of patients received thrombopoietin receptor agonist (TPO-RA) therapy, specifically eltrombopag, during the course of treatment.

### 2.1. Complications

Over the recorded follow-up period based on retrospective medical records, 20 patients (12%) developed complications. Among these, bleeding occurred in 8 patients (4.8%), infection in 6 patients (3.6%), thrombosis in 5 patients (3%), and concomitant bleeding and thrombosis in 1 patient (0.6%). The mean age of patients who developed complications was significantly higher than that of patients without complications (*p* = 0.040, Table 1).

No significant association was observed between complication development and sex or the presence of comorbidities. Bone marrow examination was performed in 51 patients. Increased megakaryocyte numbers were observed in 27 patients (52%), whereas 12 patients (23%) exhibited decreased megakaryocyte counts. Laboratory findings of patients with and without complications are summarized in Table 1. Patients with complications had significantly higher D-dimer levels compared with those without complications (*p* = 0.028). In contrast, no significant differences were observed with respect to platelet-to-lymphocyte ratio (PLR) (*p* = 0.477) or neutrophil-to-lymphocyte ratio (NLR) (*p* = 0.903). Clinical characteristics according to complication status are presented in Table 1. Patients with complications demonstrated a significantly lower bone marrow megakaryocyte production (*p* = 0.001) and a significantly higher mortality rate (*p* = 0.001). Additionally, the frequency of complications was significantly higher among patients treated with rituximab (*p* = 0.019). Additional exploratory analyses were performed to evaluate bleeding, infection, and thrombosis as separate outcomes. However, due to the limited number of events in each subgroup, no statistically significant or consistent associations were observed between inflammatory indices and individual complication types.

### 2.2. Mortality

Over the recorded follow-up period based on retrospective medical records, 11 patients (6.6%) died. The mean age of patients who died was 82.5 ± 8 years, which was significantly higher than that of survivors (*p* = 0.001). Male (*p* = 0.002) and the presence of comorbidities (*p* = 0.009) were significantly associated with increased mortality (Table 2).

Laboratory comparisons between patients with and without mortality are shown in Table 2. Patients who died had significantly lower levels of hemoglobin (*p* = 0.001), mean platelet volume (MPV) (*p* = 0.043), lymphocyte count (*p* = 0.044), and albumin (*p* = 0.002). Conversely, PLR (*p* = 0.004), total bilirubin (*p* = 0.015), direct bilirubin (*p* = 0.045), erythrocyte sedimentation rate (ESR) (*p* = 0.048), and D-dimer levels (*p* = 0.001) were significantly higher in the mortality group. Clinical characteristics according to mortality status are presented in Table 2. Notably, no mortality was observed among patients who underwent splenectomy (*p* = 0.017). The frequency of complications was significantly higher in patients who died (*p* = 0.001). Subgroup analysis revealed that bleeding (*p* = 0.004), infection (*p* = 0.007), thrombosis (*p* = 0.002), and the coexistence of bleeding and thrombosis (*p* = 0.001) were each independently associated with mortality. To further evaluate potential confounding effects, subgroup analyses were performed by stratifying patients according to age, sex, presence of comorbidities, treatment modalities, and splenectomy status. The associations between key inflammatory markers (PLR, D-dimer, and albumin) and mortality remained generally consistent across these subgroups. However, due to the limited number of events within certain strata, these findings should be interpreted with caution.

**Figure 1:**
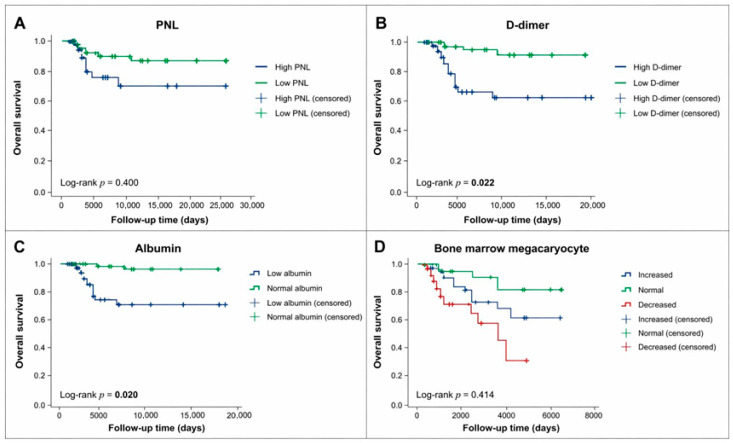
Kaplan-Meier survival curves according to biomarker-based groups. (**A**) PNL (high vs. low), (**B**) D-dimer (high vs. low), (**C**) albumin (low vs. normal), and (**D**) bone marrow megacaryocyte status (increased vs. normal vs. decreased). *p* values were calculated using the log-rank test.

Kaplan–Meier survival analyses were performed to evaluate the prognostic impact of biomarker-based groups. Patients with high D-dimer levels had significantly worse overall survival compared to those with low levels (log-rank test, χ^2^ = 5.272, *p* = 0.022). Similarly, hypoalbuminemia was associated with significantly reduced survival compared to normal/high albumin levels (log-rank test, χ^2^ = 5.406, *p* = 0.020). In contrast, no statistically significant differences in survival were observed between groups stratified by PNL (log-rank test, χ^2^ = 0.708, *p* = 0.400) or bone marrow megakaryocyte status (log-rank test, χ^2^ = 1.764, *p* = 0.414) (Figure 1).

### 2.3. Treatment Response and Relapse

No laboratory parameters were found to be significantly associated with response to steroid therapy (Table 3). Comparisons between patients who responded to intravenous immunoglobulin (IVIG) therapy and those who did not are shown in Table 3.

Patients with higher levels of total protein (*p* = 0.028), albumin (*p* = 0.031), and fibrinogen (*p* = 0.003), as well as lower ESR values (*p* = 0.001), exhibited significantly better responses to IVIG therapy. Biochemical factors associated with relapse are summarized in Table 4.

Patients who experienced relapse had significantly higher levels of reticulocytes, ESR, and urea. Clinical features associated with relapse are presented in Table 4. Relapse was significantly more frequent among patients with poor steroid response, poor IVIG response, and those who developed bleeding, infection, or thrombosis. In addition, antinuclear antibody (ANA) positivity and Helicobacter pylori antigen positivity were significantly more common in patients with relapse.

## 3. Discussion

Immune thrombocytopenic purpura (ITP) is a clinically heterogeneous autoimmune disorder characterized by immune-mediated platelet destruction and impaired platelet production, resulting in a broad spectrum of disease severity and outcomes [13,14]. In this single-center retrospective study with long-term follow-up, we evaluated inflammatory markers, bone marrow findings, treatment modalities, and clinical outcomes in adult patients with primary ITP. Our findings highlight the prognostic significance of systemic inflammation and megakaryocyte dysfunction, while also revealing several paradoxical observations that contribute to the evolving understanding of ITP pathophysiology.

### 3.1. Complications and Inflammatory Activity

Complications were observed in 12% of patients, with bleeding being the most frequent event, followed by infection and thrombosis. This distribution is consistent with previous studies demonstrating that, although bleeding remains the hallmark clinical manifestation of ITP, non-hemorrhagic complications significantly contribute to morbidity, particularly in older patients [15,16,17]. The significantly higher mean age in patients who developed complications supports earlier observations that aging is associated with immune senescence, chronic inflammation, and endothelial dysfunction, all of which may exacerbate disease severity in ITP [18]. A key finding of this study was the significantly elevated D-dimer levels in patients who developed complications. D-dimer is a well-established marker of coagulation activation and fibrinolysis and has increasingly been recognized as a surrogate marker of thromboinflammatory activity [19,20]. Although ITP is traditionally classified as a bleeding disorder, growing evidence suggests that immune-mediated platelet activation, endothelial injury, and increased platelet-derived microparticles may paradoxically promote a hypercoagulable state [19]. Our results support this emerging concept and suggest that D-dimer may serve as a clinically useful biomarker for identifying ITP patients at increased risk of complications. In contrast, neutrophil-to-lymphocyte ratio (NLR) and platelet-to-lymphocyte ratio (PLR) were not associated with complication development. This finding appears paradoxical given prior reports demonstrating the prognostic value of these indices in inflammatory and autoimmune disorders, including ITP [21,22,23,24,25,26]. A possible explanation is that acute complications in ITP may be driven predominantly by coagulation and endothelial mechanisms rather than systemic leukocyte redistribution. Additionally, the heterogeneity of complications may have reduced the discriminatory power of these indices when analyzed as a composite endpoint. Importantly, bleeding, infection, and thrombosis represent distinct clinical entities with different underlying mechanisms. In our study, these outcomes were analyzed as a composite endpoint, which may have reduced the ability of certain inflammatory indices, such as NLR and PLR, to discriminate between specific complication types. Although exploratory subgroup analyses were performed, the limited number of events in each category restricted the statistical power of these analyses. Therefore, future studies with larger patient populations are needed to evaluate outcome-specific predictors more accurately.

### 3.2. Bone Marrow Megakaryopoiesis and Prognostic Implications

Bone marrow examination revealed heterogeneous megakaryocyte patterns, consistent with earlier morphologic and kinetic studies in ITP [27,28,29,30,31]. While increased megakaryocyte numbers reflect compensatory responses to peripheral platelet destruction, reduced megakaryocyte production was significantly associated with complications, mortality, and relapse in our cohort. This observation highlights an important paradox in ITP pathophysiology. Although the disease is classically defined by peripheral platelet destruction, impaired megakaryopoiesis appears to play a critical role in determining disease severity and prognosis [28,29,30]. Experimental and clinical studies have demonstrated that autoantibodies directed against megakaryocyte antigens, cytotoxic T lymphocyte–mediated suppression, and inflammatory cytokines inhibit megakaryocyte maturation and platelet release [31]. Our findings suggest that reduced megakaryocyte production may represent an advanced stage of immune dysregulation and could serve as a marker of poor prognosis.

### 3.3. Mortality and Systemic Inflammation

It is important to note that the term “systemic inflammation” in this study reflects a chronic, baseline inflammatory milieu rather than acute inflammatory events, as all laboratory parameters were evaluated at the time of diagnosis. The mortality rate of 6.6% observed in this study is consistent with long-term population-based studies reporting increased mortality among adult patients with ITP compared with the general population [13,14,17]. Advanced age emerged as the strongest predictor of mortality, in line with previous observations linking aging to increased vulnerability to infections, thrombotic events, and treatment-related complications in ITP [16,18]. Male sex and the presence of comorbidities were also significantly associated with mortality, underscoring the cumulative impact of patient-related risk factors. Laboratory analyses revealed that patients who died had significantly lower hemoglobin, lymphocyte, and albumin levels. These findings suggest a combination of bone marrow dysfunction, immune exhaustion, and chronic inflammation, which have been associated with adverse outcomes in various inflammatory and hematologic conditions [32,33]. Given that age, male sex, and comorbidities were significantly associated with mortality in our cohort, the potential for confounding should be carefully considered. Although subgroup analyses demonstrated broadly consistent associations between inflammatory markers and mortality across clinically relevant strata, the relatively small number of events may limit the statistical power of these analyses. Therefore, residual confounding cannot be fully excluded, and future studies with larger sample sizes and multivariable modeling are warranted. Hypoalbuminemia, in particular, emerged as one of the strongest predictors of mortality. Albumin is a negative acute-phase reactant and a robust marker of systemic inflammation and nutritional status, and its prognostic value has been demonstrated across a wide range of clinical settings [34,35,36]. Conversely, elevated PLR, erythrocyte sedimentation rate (ESR), bilirubin, and D-dimer levels were significantly associated with mortality. The association between elevated PLR and mortality, despite its lack of association with complication development, represents another paradoxical finding. This may indicate that PLR reflects chronic inflammatory burden rather than acute inflammatory activity [21,22,23]. Elevated ESR and bilirubin levels may further reflect sustained systemic inflammation, subclinical hemolysis, or organ dysfunction, all of which are linked to increased mortality risk [32,37,38,39,40]. Recent advances in multi-omics approaches have significantly expanded our understanding of the complex and heterogeneous nature of immune thrombocytopenia. Integrative analyses combining transcriptomics, proteomics, and metabolomics have revealed distinct molecular signatures associated with immune dysregulation, platelet destruction, and impaired megakaryopoiesis. These studies have demonstrated that alterations in immune cell subsets, cytokine signaling pathways, and metabolic reprogramming play a central role in disease pathogenesis and progression. In particular, proteomic and metabolomic profiling of bone marrow and peripheral blood samples has identified potential biomarkers associated with disease severity and treatment response, including glucocorticoid resistance. Moreover, the integration of multi-layered omics data using machine learning approaches has enabled the identification of novel molecular targets and patient subgroups, supporting a more personalized approach to disease management. These findings are consistent with our results, which suggest that systemic inflammation and impaired megakaryocyte production are key determinants of clinical outcomes. Although our study is based on routinely available laboratory parameters rather than high-throughput omics data, the observed associations may reflect underlying molecular mechanisms identified in multi-omics studies. Therefore, simple inflammatory markers such as PLR, D-dimer, and albumin may serve as clinically accessible surrogates of complex biological processes, bridging the gap between advanced molecular profiling and real-world clinical practice. Future studies integrating clinical data with multi-omics platforms are warranted to further refine prognostic models and enable precision medicine strategies in ITP [41].

### 3.4. Treatment Response and Clinical Outcomes

No laboratory parameters were found to predict response to corticosteroid therapy, consistent with previous reports highlighting the unpredictable nature of steroid responsiveness in ITP [42]. Steroid resistance likely reflects complex immunological mechanisms, including Fc receptor polymorphisms, autoreactive T-cell activity, and cytokine-mediated immune dysregulation, which are not captured by routine laboratory testing. In contrast, response to intravenous immunoglobulin (IVIG) therapy was significantly associated with higher total protein, albumin, and fibrinogen levels and lower ESR values. These findings suggest that patients with preserved nutritional status and lower baseline inflammatory activity are more likely to benefit from IVIG therapy, which exerts its effects through immune modulation and Fc receptor blockade. Notably, no mortality was observed among patients who underwent splenectomy. Although splenectomy carries long-term infectious risks, it remains one of the most effective therapeutic options for refractory ITP and has been shown to provide durable remission and survival benefits in carefully selected patients [43,44].

### 3.5. Relapse and Immune Reactivation

Relapse was significantly associated with elevated reticulocyte count, ESR, and urea levels, suggesting ongoing inflammatory activity and metabolic stress. Clinically, relapse was more frequent among patients with poor responses to steroids and IVIG, as well as those who developed bleeding, infection, or thrombosis. These findings support the concept that relapse represents immune reactivation rather than a transient fluctuation in platelet count. Furthermore, higher rates of antinuclear antibody (ANA) positivity and Helicobacter pylori antigen positivity were observed among relapsed patients, reinforcing the role of autoimmune predisposition and chronic infection in disease persistence and recurrence [43,45].

In the present study, receiver operating characteristic (ROC) and correlation analyses were performed to further explore the relationships between inflammatory markers and clinical outcomes. However, these findings were not emphasized in detail, as the overall discriminatory performance of the evaluated markers was modest and did not provide additional clinically meaningful thresholds beyond group-based comparisons. In particular, the area under the curve (AUC) values for key parameters such as PLR and D-dimer remained within a limited range, suggesting restricted predictive accuracy when used in isolation. Similarly, correlation analyses demonstrated weak-to-moderate associations, indicating that these biomarkers likely reflect complex and multifactorial biological processes rather than direct linear relationships with clinical endpoints. Another important consideration is the presence of variability in several laboratory parameters. Some variables exhibited wide standard deviations and fluctuating values across groups, which may reflect non-normal data distribution, potential outliers, or underlying biological heterogeneity inherent to ITP. Given the retrospective design and real-world nature of the dataset, extreme values and missing data points could not be entirely excluded. To minimize potential bias, appropriate non-parametric statistical methods were applied where necessary. Nevertheless, such variability may have attenuated the strength of statistical associations and should be taken into account when interpreting the findings. Therefore, the results of this study should be interpreted with caution, particularly regarding the predictive utility of individual biomarkers, and future prospective studies with standardized measurements and larger sample sizes are warranted to validate these observations.

**Limitations**

This study has several limitations. First, its retrospective and single-center design may limit the generalizability of the findings and introduce the potential for selection bias. Second, all data were derived from electronic medical records, and some variables may have been subject to incomplete or inconsistent documentation. Third, inflammatory markers were evaluated only at baseline, and dynamic changes over time were not assessed. Fourth, although we analyzed multiple clinical and laboratory parameters, residual confounding factors cannot be fully excluded. Finally, the relatively small number of mortality events may have limited the statistical power for certain subgroup analyses. Therefore, our findings should be interpreted with caution, and prospective, multicenter studies are needed to validate these results. Furthermore, complications were analyzed as a composite outcome, and the relatively small number of bleeding, infection, and thrombosis events limited the ability to perform robust outcome-specific analyses. In addition, although subgroup analyses were performed to explore potential confounding effects, the relatively small number of mortality events limited the robustness of these stratified evaluations.

## 4. Materials and Methods

This study involved the analysis of electronic medical records of patients diagnosed with immune thrombocytopenic purpura (ITP) who presented to Muğla Training and Research Hospital between January 2015 and December 2024. This single-center study was reviewed and approved by the Ethics Committee of Muğla Sıtkı Koçman University Faculty of Medicine (approval number: 250188/217; approval date: 11 March 2026) and was conducted in accordance with the ethical principles of the Declaration of Helsinki and its subsequent amendments. Demographic characteristics, treatment modalities, treatment responses, survival outcomes, and laboratory findings at the time of diagnosis were comprehensively evaluated. This was a retrospective observational study, and no direct patient contact or prospective follow-up was conducted. All data were obtained from electronic medical records.

### 4.1. Study Population

Primary ITP was defined as a platelet count ≤ 100 × 10^9^/L in the absence of evidence for other causes of thrombocytopenia. Secondary causes of thrombocytopenia—including sepsis, drug-induced thrombocytopenia, hematological disorders, portal hypertension, and rheumatologic diseases—were excluded. Inclusion criteria comprised all patients aged 18 years and older diagnosed with primary ITP. Exclusion criteria included patients younger than 18 years, those who became pregnant during ITP follow-up, and breastfeeding individuals. Thrombopoietin receptor agonist (TPO-RA) therapy, specifically eltrombopag, was administered to a subset of patients according to clinical indications.

### 4.2. Statistical Analysis

Statistical analyses were performed using IBM SPSS Statistics software (Statistical Package for the Social Sciences version 22, IBM Corp., Armonk, NY, USA). Continuous variables were assessed for normality using the Kolmogorov–Smirnov test and by visual inspection of histograms. Normally distributed continuous variables were expressed as mean ± standard deviation (SD), whereas non-normally distributed variables were presented as median (interquartile range, IQR). Categorical variables were summarized as frequencies and percentages. Comparisons between two independent groups were conducted using Student’s *t*-test for normally distributed variables and the *Mann–Whitney U* test for non-normally distributed variables. For comparisons involving more than two groups, one-way analysis of variance (ANOVA) or the *Kruskal–Wallis* test was applied, as appropriate. Categorical variables were compared using the *Chi-square* test or *Fisher’s* exact test, depending on expected cell counts. Inflammatory indices derived from complete blood count parameters—including the neutrophil-to-lymphocyte ratio (NLR), platelet-to-lymphocyte ratio (PLR), and platelet-to-neutrophil ratio (PNR)—were calculated and analyzed as markers of systemic inflammation. Correlations between inflammatory indices and clinical or laboratory parameters were assessed using Pearson’s correlation coefficient for normally distributed data or Spearman’s rank correlation coefficient for non-normally distributed data. Where appropriate, receiver operating characteristic (ROC) curve analysis was performed to evaluate the predictive value of inflammatory indices for clinical outcomes, and the area under the curve (AUC) was calculated. Due to the limited sample size, continuous variables were dichotomized at their median values, and Kaplan–Meier survival analyses were performed instead of determining optimal cut-off points using ROC curve analysis.

Optimal cut-off values were determined using the *Youden* index. All statistical tests were two-tailed, and a *p*-value < 0.05 was considered statistically significant. Correlation analyses were performed specifically to evaluate relationships between inflammatory indices and continuous clinical or laboratory parameters, and statistically significant correlations were interpreted separately from group-based associations.

## 5. Conclusions

**Figure 2:**
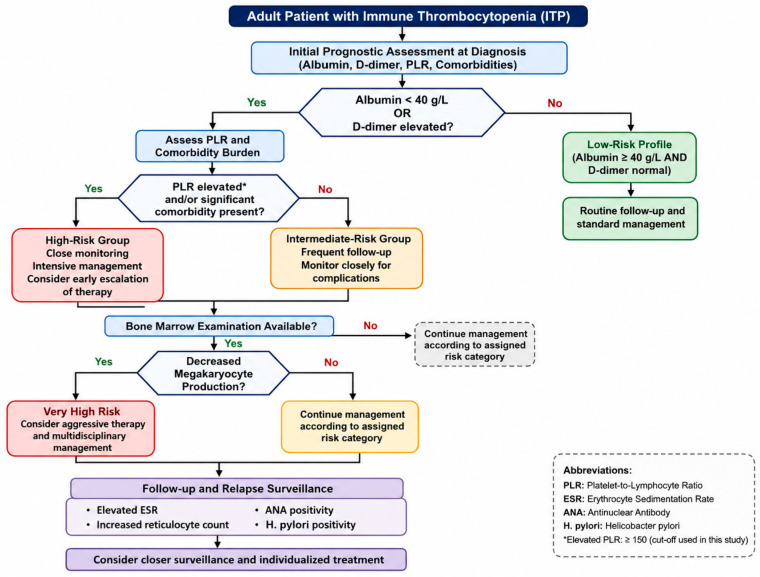
Clinical Decision-Making Algorithm for ITP Patients.

In conclusion, this study demonstrates that systemic inflammation and bone marrow megakaryocyte dysfunction play central roles in determining clinical outcomes in patients with immune thrombocytopenia. While commonly used inflammatory indices such as NLR and PLR showed limited utility in predicting complications, markers including PLR, D-dimer, and serum albumin were significantly associated with mortality and disease severity. Accordingly, we have developed a decision-making algorithm based on key prognostic markers identified in our analysis, including albumin, D-dimer, PLR, and bone marrow megakaryocyte status (Figure 2). This flowchart can be used as a new figure to facilitate clinical interpretation and support risk stratification in patients with ITP. Reduced megakaryocyte production emerged as an important indicator of poor prognosis, underscoring the contribution of impaired thrombopoiesis alongside peripheral platelet destruction. Additionally, treatment responses and relapse patterns appeared to be influenced by underlying inflammatory and immunological activity. Although several studies have evaluated individual inflammatory markers in immune thrombocytopenia, most have focused on isolated parameters without integrating them with comprehensive clinical outcomes. The present study provides a broader perspective by simultaneously analyzing multiple hematological inflammatory indices together with clinically relevant endpoints, including complications, mortality, treatment response, and relapse in a relatively large real-world cohort with extended follow-up. This integrative approach allows for a more clinically meaningful interpretation of inflammatory biomarkers in ITP. Moreover, our findings suggest that traditional indices such as NLR and PLR may have limited predictive utility for acute complications, whereas PLR, D-dimer, and albumin appear to have stronger associations with mortality and overall prognosis. In addition, the combined evaluation of systemic inflammation and bone marrow megakaryocyte activity offers a more comprehensive understanding of disease severity, which has not been sufficiently emphasized in previous studies. Taken together, these findings suggest that readily available laboratory parameters may provide valuable, cost-effective tools for risk stratification and personalized management of ITP patients, although further prospective and mechanistic studies are warranted to validate these observations. 

## Figures and Tables

**Table 1:** Clinical outcomes, demographic characteristics, laboratory findings, and treatment-related factors associated with complications in patients with ITP.

**Outcome/Complication**	***n***		**%**	
Mortality	20		12	
Bleeding	8		4.8	
Infection	6		3.6	
Thrombosis	5		3	
Bleeding + Thrombosis	1		0.6	
Variable	Complications (+) (*n* = 20)	Complications (−) (*n* = 146)		*p*-value
Age (years)	64.4 ± 23.8	52.3 ± 17.3		0.04 *
Gender (Male/Female)	9/11 (45%/55%)	53/93 (36.3%/63.7%)		0.451
Comorbidity, *n* (%)	12 (60%)	76 (52.1%)		0.504
Laboratory Parameters	Complications (+) (*n* = 20)	Complications (−) (*n* = 146)		*p*-value
Hemoglobin (g/dL)	11.9 ± 2.2	12.9 ± 2.2		0.054
Mean corpuscular volume (MCV, fL)	85.2 ± 7.4	89.5 ± 66.7		0.77
Mean platelet volume (MPV, fL)	5.84 ± 5.19	9.14 ± 5.18		0.06
White blood cell (WBC) count	7008 ± 2982	7878 ± 3422		0.281
Neutrophil count	4705 ± 2931	5275 ± 3199		0.452
Neutrophil percentage (%)	63.5 ± 15.5	63.2 ± 15.8		0.936
Lymphocyte count	1607.5 ± 840.8	1914 ± 949		0.172
Monocyte count	448.5 ± 195.7	541 ± 282		0.158
Eosinophil count	190.4 ± 170.5	134.7 ± 131.1		0.283
Platelet count	66,976 ± 33,350	48,331 ± 32,452		0.941
Lymphocyte-to-monocyte ratio (LMR)	8.82 ± 5.61	4.35 ± 2.81		0.188
Neutrophil-to-lymphocyte ratio (NLR)	3.98 ± 3.89	4.89 ± 4.03		0.903
Eosinophil-to-lymphocyte ratio (ELR)	0.14 ± 0.12	0.11 ± 0.08		0.097
Platelet-to-lymphocyte ratio (PLR)	107.4 ± 35	23.3 ± 17.6		0.477
Lactate dehydrogenase-to-lymphocyte ratio (LDH/Lymphocyte)	0.18 ± 0.11	0.16 ± 0.14		0.585
Neutrophil percentage-to-albumin ratio	1.69 ± 0.56	1.73 ± 1.51		0.891
Albumin-to-fibrinogen ratio (AFR)	0.14 ± 0.06	0.16 ± 0.08		0.249
Glucose (mg/dL)	121.2 ± 24.8	119.2 ± 50.7		0.862
Alanine aminotransferase (ALT, U/L)	14.6 ± 6.8	37.7 ± 25.2		0.209
Aspartate aminotransferase (AST, U/L)	16.6 ± 4.4	22.8 ± 21.6		0.206
Gamma-glutamyl transferase (GGT, U/L)	24 ± 22.6	37.3 ± 24.4		0.967
Lactate dehydrogenase (LDH, U/L)	226.9 ± 53.4	230 ± 105		0.893
Alkaline phosphatase (ALP, U/L)	73.8 ± 26.7	78.7 ± 43.4		0.641
Urea (mg/dL)	36.1 ± 17.8	34 ± 19.5		0.656
Creatinine (mg/dL)	0.80 ± 0.26	0.78 ± 0.28		0.78
Uric acid (mg/dL)	5.1 ± 1.8	4.8 ± 1.5		0.425
Total protein (g/dL)	71.7 ± 9.4	72.1 ± 8.5		0.836
Albumin (g/dL)	39.0 ± 6.6	41.9 ± 7.4		0.102
Total bilirubin (mg/dL)	0.73 ± 0.47	0.55 ± 0.4		0.062
Direct bilirubin (mg/dL)	0.28 ± 0.18	0.21 ± 0.19		0.12
Total cholesterol (mg/dL)	191.4 ± 50.2	177.6 ± 48.2		0.41
Triglycerides (mg/dL)	129.9 ± 43.8	135.1 ± 81.7		0.851
Erythrocyte sedimentation rate (ESR, mm/h)	29.8 ± 27.8	24.8 ± 21.9		0.209
C-reactive protein (CRP, mg/dL)	21.5 ± 12	11.6 ± 6.8		0.094
D-dimer (mg/L)	1597.8 ± 1303.6	1213 ± 790		0.028 *
Fibrinogen (mg/dL)	319.5 ± 95.9	311 ± 204		0.87
Reticulocyte (%)	1.63 ± 1.08	7.70 ± 1.47		0.935
Ferritin (ng/mL)	198.4 ± 152.6	203.2 ± 98.2		0.275
Indirect Coombs test	4 (20%)	18 (12.3%)		0.343
Antinuclear antibody (ANA)	3 (15%)	19 (13%)		0.806
Helicobacter pylori antigen	1 (5%)	7 (4.8%)		0.968
Clinical Characteristics	Complications (+) (*n* = 20)	Complications (−) (*n* = 146)		*p*-value
Increased bone marrow megakaryocyte count	5 (25%)	22 (15.1%)		0.259
Decreased bone marrow megakaryocyte count	5 (25%)	7 (4.8%)		0.001 *
Steroid therapy	19 (95%)	125 (85.6%)		0.246
Response to steroid therapy	14 (70%)	71 (48.6%)		0.071
Steroid + intravenous immunoglobulin (IVIG) therapy	15 (75%)	76 (52.1%)		0.053
Response to IVIG therapy	11 (55%)	60 (41.1%)		0.239
Eltrombopag therapy	5 (25%)	44 (30.1%)		0.637
Rituximab therapy	6 (30%)	16 (11%)		0.019 *
Splenectomy	4 (20%)	50 (34.2%)		0.202
Duration of remission (days)	8 ± 7.6	2.4 ± 1.7		0.283
Follow-up duration (days)	2014 ± 1364	3276 ± 2594		0.448
Mortality	8 (40%)	3 (2.1%)		0.001 *

Values are expressed as mean ± standard deviation or *n* (%), as appropriate. *p*-values were calculated using Student’s *t*-test or *Mann–Whitney U* test for continuous variables and Chi-square or Fisher’s exact test for categorical variables. A *p* value < 0.05 was considered statistically significant. Statistically significant results are indicated with an asterisk (*).

**Table 2:** Clinical outcomes, demographic characteristics, laboratory findings, and treatment-related factors associated with mortality in patients with ITP.

**Variable**	**Mortality (+) (*n* = 11)**	**Mortality (−) (*n* = 155)**	***p*-Value**
Age (years)	82.5 ± 8	51.7 ± 17.4	0.001 *
Gender (Male/Female)	9/2 (81.8%/18.2%)	53/102 (34.2%/65.8%)	0.002 *
Comorbidity, *n* (%)	10 (90.9%)	78 (50.3%)	0.009 *
Laboratory Parameters	Mortality (+) (*n* = 11)	Mortality (−) (*n* = 155)	*p*-value
Hemoglobin (g/dL)	9.9 ± 2.2	13 ± 2.1	0.001 *
Mean corpuscular volume (MCV, fL)	86.8 ± 9.9	89.2 ± 64.7	0.905
Mean platelet volume (MPV, fL)	4.61 ± 4.51	8.96 ± 5.24	0.043 *
White blood cell (WBC) count	5966 ± 2923	7902 ± 3377	0.066
Neutrophil count	3905 ± 2430	5299 ± 3196	0.159
Neutrophil percentage (%)	63.5 ± 11.5	63.2 ± 16	0.939
Lymphocyte count	1327 ± 532	1916 ± 951	0.044 *
Monocyte count	576 ± 344	526 ± 269	0.560
Eosinophil count	248 ± 217	136 ± 128	0.054
Platelet count	89,402 ± 45,182	47,135 ± 31,665	0.394
Lymphocyte-to-monocyte ratio (LMR)	2.64 ± 1.08	4.64 ± 4.11	0.111
Neutrophil-to-lymphocyte ratio (NLR)	3.17 ± 2	4.9 ± 4.08	0.544
Eosinophil-to-lymphocyte ratio (ELR)	0.18 ± 0.17	0.11 ± 0.08	0.082
Platelet-to-lymphocyte ratio (PLR)	144.2 ± 55.7	22.8 ± 17.1	0.004 *
Lactate dehydrogenase-to-lymphocyte ratio (LDH/Lymphocyte)	0.2 ± 0.09	0.16 ± 0.13	0.368
Neutrophil percentage-to-albumin ratio	1.87 ± 0.54	1.72 ± 1.48	0.739
Albumin-to-fibrinogen ratio (AFR)	0.11 ± 0.04	0.16 ± 0.08	0.096
Glucose (mg/dL)	122 ± 27.5	119.2 ± 49.5	0.869
Alanine aminotransferase (ALT, U/L)	13.6 ± 5.7	36.7 ± 24.7	0.316
Aspartate aminotransferase (AST, U/L)	17.6 ± 5.5	22.3 ± 21	0.454
Gamma-glutamyl transferase (GGT, U/L)	30 ± 19.8	36.2 ± 24	0.606
Lactate dehydrogenase (LDH, U/L)	233 ± 67.6	230 ± 102	0.914
Alkaline phosphatase (ALP, U/L)	91.8 ± 44.6	77.3 ± 41.7	0.314
Urea (mg/dL)	45.5 ± 23.5	33.5 ± 18.7	0.718
Creatinine (mg/dL)	0.89 ± 0.32	0.78 ± 0.27	0.175
Uric acid (mg/dL)	4.73 ± 2.09	4.82 ± 1.54	0.862
Total protein (g/dL)	71.2 ± 9.6	72.1 ± 8.5	0.732
Albumin (g/dL)	35.1 ± 5.3	42 ± 7.2	0.002 *
Total bilirubin (mg/dL)	0.86 ± 0.54	0.55 ± 0.4	0.015 *
Direct bilirubin (mg/dL)	0.33 ± 0.2	0.21 ± 0.19	0.045 *
Total cholesterol (mg/dL)	164.5 ± 63.7	179 ± 48	0.554
Triglycerides (mg/dL)	113.3 ± 56.7	135 ± 80.1	0.585
Erythrocyte sedimentation rate (ESR, mm/h)	38.9 ± 29.1	24.7 ± 21.8	0.048 *
C-reactive protein (CRP, mg/dL)	28 ± 18.1	22.1 ± 8.1	0.154
D-dimer (mg/L)	2662 ± 2462	990 ± 746	0.001 *
Fibrinogen (mg/dL)	331 ± 114	311 ± 198	0.793
Reticulocyte (%)	2.56 ± 1.24	7.44 ± 1.43	0.712
Ferritin (ng/mL)	262 ± 184	198 ± 99.4	0.203
Indirect Coombs test	3 (27.3%)	19 (12.3%)	0.018 *
Antinuclear antibody (ANA)	2 (18.2%)	20 (12.9%)	0.073
Helicobacter pylori antigen	1 (9.1%)	7 (4.5%)	0.058
Clinical Characteristics	Mortality (+) (*n* = 11)	Mortality (−) (*n* = 155)	*p*-value
Increased bone marrow megakaryocyte count	4 (36.4%)	23 (14.8%)	0.062
Decreased bone marrow megakaryocyte count	3 (27.3%)	9 (5.8%)	0.008 *
Steroid therapy	11 (100%)	133 (85.8%)	0.18
Response to steroid therapy	7 (63.6%)	78 (50.3%)	0.393
Steroid + intravenous immunoglobulin (IVIG) therapy	8 (72.7%)	83 (53.5%)	0.217
Response to IVIG therapy	5 (45.5%)	66 (42.6%)	0.852
Eltrombopag therapy	3 (27.3%)	46 (29.7%)	0.866
Rituximab therapy	2 (18.2%)	20 (12.9%)	0.618
Splenectomy	0 (0%)	54 (34.8%)	0.017 *
Complications	8 (72.7%)	12 (7.7%)	0.001 *
Bleeding	3 (27.3%)	5 (3.2%)	0.004 *
Infection	2 (18.2%)	4 (2.6%)	0.007 *
Thrombosis	2 (18.2%)	3 (1.9%)	0.002 *
Bleeding + Thrombosis	1 (9.1%)	0 (0%)	0.001 *
Duration of remission (days)	-	3.4 ± 2.2	-
Follow-up duration (days)	2141 ± 721	3209 ± 2550	0.689

Values are expressed as mean ± standard deviation or *n* (%), as appropriate. *p*-values were calculated using Student’s *t*-test or *Mann–Whitney U* test for continuous variables and Chi-square or Fisher’s exact test for categorical variables. A *p* value < 0.05 was considered statistically significant. Statistically significant results are indicated with an asterisk (*).

**Table 3:** Laboratory parameters and clinical outcomes associated with response to steroid and intravenous immunoglobulin (IVIG) therapy in patients with ITP.

Laboratory Parameters	Response to Steroid Therapy (+) (*n* = 85)	Response to Steroid Therapy (−) (*n* = 38)	*p*-Value	Response to IVIG Therapy (+) (*n* = 71)	Response to IVIG Therapy (−) (*n* = 10)	*p*-Value
Hemoglobin (g/dL)	12.6 ± 2.1	12.6 ± 2.3	0.946	12.5 ± 2.1	11.4 ± 3.8	0.426
Mean corpuscular volume (MCV, fL)	83.3 ± 7.4	106 ± 29.5	0.289	82.7 ± 7.4	83.9 ± 8.3	0.618
Mean platelet volume (MPV, fL)	8.12 ± 5.5	5.53 ± 5.26	0.295	7.15 ± 5.48	5.87 ± 5.15	0.627
White blood cell (WBC) count	7524.6 ± 3083	8310 ± 3627	0.22	76,867 ± 3330	7800 ± 4270	0.923
Neutrophil count	5202 ± 3039	5625 ± 2894	0.471	5309 ± 3344	5359 ± 3121	0.964
Neutrophil percentage (%)	64.19 ± 15.62	66.1 ± 16.52	0.545	63.5 ± 16.8	66.30 ± 16.81	0.829
Lymphocyte count	1770 ± 829	1835 ± 1237	0.769	1788 ± 851	1770 ± 1389	0.954
Monocyte count	506 ± 267	545 ± 343	0.496	481 ± 211	498 ± 390	0.835
Eosinophil count	120 ± 117	169 ± 145	0.34	127 ± 117	280 ± 228	0.285
Platelet count	52,580 ± 30,011	32,251 ± 20,500	0.305	47,977 ± 25,211	13,864 ± 10,000	0.324
Lymphocyte-to-monocyte ratio (LMR)	5.2 ± 4.84	4.1 ± 2.33	0.398	5.16 ± 4.74	4.18 ± 2.08	0.737
Neutrophil-to-lymphocyte ratio (NLR)	5.36 ± 4.3	4.7 ± 4.41	0.688	5.99 ± 4.62	4.6 ± 4.56	0.993
Eosinophil-to-lymphocyte ratio (ELR)	0.1 ± 0.07	0.11 ± 0.09	0.436	0.11 ± 0.08	0.18 ± 0.13	0.219
Platelet-to-lymphocyte ratio (PLR)	54.6 ± 20.5	24.83 ± 13.43	0.446	25.9 ± 15	9.22 ± 6.86	0.328
LDH/Lymphocyte	0.17 ± 0.15	0.19 ± 0.13	0.596	0.17 ± 0.15	0.19 ± 0.11	0.754
Neutrophil percentage-to-albumin ratio	1.82 ± 1.63	1.64 ± 0.55	0.518	1.85 ± 1.78	1.8 ± 0.56	0.929
Albumin-to-fibrinogen ratio (AFR)	0.16 ± 0.08	0.15 ± 0.06	0.892	0.15 ± 0.07	0.14 ± 0.05	0.798
Glucose (mg/dL)	116 ± 30.5	135.6 ± 71.4	0.11	119.7 ± 47.6	157 ± 92.2	0.244
Alanine aminotransferase (ALT, U/L)	23.3 ± 21.4	33 ± 24.5	0.53	20.1 ± 18.8	32.3 ± 15.9	0.53
Aspartate aminotransferase (AST, U/L)	20.8 ± 13.2	21.2 ± 17.6	0.88	19 ± 8.7	21.1 ± 15.5	0.516
Gamma-glutamyl transferase (GGT, U/L)	32.6 ± 25	48.1 ± 27.5	0.74	21.4 ± 17.9	25.7 ± 22.3	0.889
Lactate dehydrogenase (LDH, U/L)	224.3 ± 69.6	233.2 ± 54.9	0.487	220 ± 59.7	244 ± 80.8	0.261
Alkaline phosphatase (ALP, U/L)	79.9 ± 31.8	70.9 ± 20.5	0.068	71.1 ± 23.7	69.7 ± 15.3	0.854
Urea (mg/dL)	36. ± 20.9	33.9 ± 15.8	0.584	32.6 ± 16.8	46.9 ± 32.7	0.206
Creatinine (mg/dL)	1.9 ± 0.89	0.83 ± 0.24	0.464	0.89 ± 0.78	1.06 ± 0.63	0.503
Uric acid (mg/dL)	4.84 ± 1.57	4.99 ± 1.73	0.643	4.76 ± 1.49	5.97 ± 1.78	0.028 *
Total protein (g/dL)	72.3 ± 9.1	71.3 ± 8	0.583	74.7 ± 9.1	67.9 ± 9.1	0.031 *
Albumin (g/dL)	40.9 ± 7.8	41.5 ± 5.6	0.697	40.8 ± 8.2	37.5 ± 4.3	0.216
Total bilirubin (mg/dL)	0.9 ± 0.61	0.64 ± 0.44	0.853	0.57 ± 0.37	0.53 ± 0.34	0.768
Direct bilirubin (mg/dL)	0.89 ± 0.29	0.24 ± 0.14	0.704	0.21 ± 0.12	0.2 ± 0.12	0.694
Total cholesterol (mg/dL)	186 ± 55.2	173.7 ± 48.1	0.299	173 ± 48	185.6 ± 51.1	0.467
Triglycerides (mg/dL)	143 ± 86.6	196 ± 41.7	0.37	132 ± 68.2	404 ± 80.9	0.001 *
Erythrocyte sedimentation rate (ESR, mm/h)	24.6 ± 23.7	28.5 ± 24.3	0.951	30 ± 28.1	42 ± 31.2	0.784
C-reactive protein (CRP, mg/dL)	29.3 ± 10.6	10.8 ± 7.1	0.477	9.88 ± 2.94	9.08 ± 6.32	0.706
D-dimer (mg/L)	1093.5 ± 916.5	1727 ± 1111	0.536	973 ± 888	2484 ± 1462	0.003 *
Fibrinogen (mg/dL)	302 ± 95.5	349 ± 33	0.298	340 ± 271	282 ± 83.5	0.511
Reticulocyte (%)	2.62 ± 1.28	0.99 ± 0.42	0.224	1.5 ± 1.08	1.41 ± 0.69	0.462
Ferritin (ng/mL)	88.7 ± 42	125 ± 79.6	0.244	84.2 ± 10.9	66.5 ± 6.5	0.619

Values are expressed as mean ± standard deviation or *n* (%), as appropriate. *p*-values were calculated using Student’s *t*-test or *Mann–Whitney U* test for continuous variables and Chi-square or Fisher’s exact test for categorical variables. A *p* value < 0.05 was considered statistically significant. Statistically significant results are indicated with an asterisk (*).

**Table 4:** Clinical outcomes, demographic characteristics, laboratory findings, and treatment-related factors according to relapse status in patients with ITP.

**Variable**	**Relapse (+) (*n* = 127)**	**Relapse (−) (*n* = 39)**	***p*-Value**
Gender (Male/Female)	48/79 (77.4%/76%)	14/25 (22.6%/24%)	0.83
Comorbidity, *n* (%)	65 (73.9%)	23 (26.1%)	0.394
Laboratory Parameters	Relapse (+) (*n* = 127)	Relapse (−) (*n* = 39)	*p*-value
Hemoglobin (g/dL)	12.6 ± 2.1	13.0 ± 2.4	0.239
Mean corpuscular volume (MCV, fL)	84.4 ± 7.6	83.9 ± 8.3	0.486
Mean platelet volume (MPV, fL)	8.44 ± 5.3	6.40 ± 7.63	0.659
Neutrophil count	5177 ± 324	5303.0 ± 2926.7	0.604
Neutrophil percentage (%)	63.9 ± 15.9	62.96 ± 15.17	0.836
Monocyte count	541 ± 272	492.0 ± 279.7	0.221
Eosinophil count	134 ± 140	154.87 ± 139.20	0.215
Neutrophil-to-lymphocyte ratio (NLR)	3.97 ± 4.51	4.16 ± 5.59	0.742
Eosinophil-to-lymphocyte ratio (ELR)	0.07 ± 0.08	0.10 ± 0.18	0.57
Neutrophil percentage-to-albumin ratio	1.79 ± 1.61	1.50 ± 0.46	0.439
Albumin-to-fibrinogen ratio (AFR)	0.15 ± 0.07	0.16 ± 0.07	0.181
Glucose (mg/dL)	117 ± 45.1	128.2 ± 56.8	0.34
Alanine aminotransferase (ALT, U/L)	24.2 ± 37.1	23.18 ± 30.14	0.79
Aspartate aminotransferase (AST, U/L)	22.7 ± 22.5	21.1 ± 11.1	0.221
Gamma-glutamyl transferase (GGT, U/L)	25.6 ± 40	19.9 ± 15.8	0.989
Lactate dehydrogenase (LDH, U/L)	231 ± 109	226.6 ± 61.8	0.566
Urea (mg/dL)	23.3 ± 48.1	7.6 ± 9.5	0.001 *
Creatinine (mg/dL)	1.53 ± 7.33	0.79 ± 0.35	0.622
Uric acid (mg/dL)	4.74 ± 1.56	5.04 ± 1.59	0.407
Albumin (g/dL)	42.5 ± 7.9	42.5 ± 4.4	0.697
Direct bilirubin (mg/dL)	0.29 ± 0.7	0.19 ± 0.76	0.898
Erythrocyte sedimentation rate (ESR, mm/h)	23.9 ± 24.9	19.3 ± 25.8	0.043 *
C-reactive protein (CRP, mg/dL)	9.63 ± 24.8	5.75 ± 12.40	0.26
D-dimer (mg/L)	1006 ± 1422	610.3 ± 621.0	0.468
Fibrinogen (mg/dL)	319 ± 214	296.2 ± 130.9	0.415
Reticulocyte (%)	2 ± 8.4	0.09 ± 0.15	0.001 *
Ferritin (ng/mL)	115 ± 229	75.4 ± 73.9	0.935
Antinuclear antibody (ANA)	19 (86.4%)	3 (13.6%)	0.001 *
Helicobacter pylori antigen	7 (87.5%)	1 (12.5%)	0.016 *
Clinical Characteristics	Relapse (+) (*n* = 127)	Relapse (−) (*n* = 39)	*p*-value
Increased bone marrow megakaryocyte count	24 (88.9%)	3 (11.1%)	0.051
Response to steroid therapy	81 (95.3%)	4 (4.7%)	0.001 *
Response to IVIG therapy	54 (76.1%)	17 (23.9%)	0.003 *
Splenectomy	15 (27.8%)	39 (72.2%)	0.339
Complications	17 (85%)	3 (15%)	0.444
Bleeding	7 (87.5%)	1 (12.5%)	0.007 *
Infection	4 (66.7%)	2 (33.3%)	0.002 *
Thrombosis	5 (100%)	0 (0%)	0.001 *
Bleeding + Thrombosis	1 (100%)	0 (0%)	0.689
Mortality	11 (100%)	0 (0%)	0.057

Values are expressed as mean ± standard deviation or *n* (%), as appropriate. *p*-values were calculated using Student’s *t*-test or *Mann–Whitney U* test for continuous variables and Chi-square or Fisher’s exact test for categorical variables. A *p* value < 0.05 was considered statistically significant. Statistically significant results are indicated with an asterisk (*).

## Data Availability

The data that support the findings of this study are available from the corresponding author upon reasonable request.
